# Plasma resistant atypical hemolytic uremic syndrome associated with a *CFH* mutation treated with eculizumab: a case report

**DOI:** 10.1186/s13256-015-0575-y

**Published:** 2015-04-29

**Authors:** Mustafa Sevinc, Taner Basturk, Tuncay Sahutoglu, Tamer Sakaci, Yener Koc, Elbis Ahbap, Cuneyt Akgol, Ekrem Kara, Vicky Brocklebank, Tim HJ Goodship, David Kavanagh, Abdulkadir Unsal

**Affiliations:** 1 Department of Nephrology, Sisli Hamidiye Etfal Training and Education Hospital, Halaskargazi Cad. Etfal Sok, 34371 Şişli, Istanbul Turkey; 2 Institute of Genetic Medicine, Newcastle University, Newcastle upon Tyne, UK

**Keywords:** Eculizumab, Factor H, Complement, Hemolytic uremic syndrome

## Abstract

**Introduction:**

Thrombotic microangiopathies are a group of diseases presenting as microangiopathic hemolytic anemia, thrombocytopenia and end-organ dysfunction. As the role of the complement system was elucidated in atypical hemolytic uremic syndrome pathogenesis, eculizumab was successfully introduced into clinical practice. We present a large pedigree with multiple individuals carrying a functionally significant novel factor H mutation. We describe the proband’s presentation following a presumed infectious trigger requiring plasma exchange and hemodialysis.

**Case presentation:**

A 32-year-old Caucasian woman presented with pyrexia and headache lasting one week to our Emergency Department. She gave no history of diarrhea or other symptoms to account for her high temperature. She was not taking any medication. She was pyrexial *(38°C),* tachycardic *(110bpm)* and hypertensive *(160/110mmHg).* Her fundoscopy revealed grade IV hypertensive retinopathy. She had mild pretibial and periorbital edema, with oliguria (450mL/day). She had a pregnancy one year previously, during which she had hypertension, proteinuria and edema, with successful delivery at term. Her mother had died in her early 30s with a clinical picture consistent with thrombotic microangiopathy. Her laboratory evaluation showed microangiopathic hemolytic anemia. After 22 sessions of plasma exchange, her lactate dehydrogenase levels started to climb. As a result, she was classified as plasma resistant and eculizumab therapy was instituted. Her lactate dehydrogenase level and platelet count normalized, and her renal function recovered after three months of dialysis.

**Conclusions:**

We demonstrate that, even in patients with atypical hemolytic uremic syndrome and prolonged dialysis dependence, recovery of renal function can be seen with eculizumab treatment. We suggest a treatment regime of at least three months prior to evaluation of efficacy.

## Introduction

Thrombotic microangiopathies (TMAs) are a group of conditions characterized by microangiopathic hemolytic anemia, thrombocytopenia and microvascular occlusion. These include thrombotic thrombocytopenic purpura (TTP), Shiga toxin-associated hemolytic uremic syndrome, disseminated intravascular coagulation, secondary TMAs (such as those caused by drugs and malignancy) and atypical hemolytic uremic syndrome (aHUS).

The last 20 years has seen the elucidation of the pathogenesis of these conditions but, until recently, this had no bearing on the treatment of disease [1]. TTP, an acquired or congenital deficiency of ADAMTS13, and aHUS, an over-activation of the alternative complement pathway (most commonly inherited), were both treated with plasma exchange.

More recently eculizumab, a recombinant monoclonal antibody directed against complement component C5, has been successfully used in the management of aHUS [2]. A non-randomized uncontrolled trial has suggested improved efficacy over plasma exchange [3]. Despite its expense, it has revolutionized the management of aHUS, but has necessitated the rapid differentiation of aHUS from ADAMTS13 deficiency to allow its use in a timely manner. Without plasma exchange TTP can be rapidly fatal, and the clinician needs to be certain that a deficiency of ADAMTS13 is not the cause of the TMA before plasma exchange can be withdrawn and eculizumab is initiated.

We demonstrate a functionally significant complement factor H (*CFH) genetic* mutation in the proband and demonstrate incomplete penetrance in her family. We describe the clinical course, with plasma resistance necessitating the introduction of eculizumab. We show that even following prolonged dialysis dependence, eculizumab can prevent the TMA process, allowing renal recovery and dialysis independence.

## Case presentation

A 32-year-old Caucasian woman presented with pyrexia and headache lasting one week to our Emergency Department. She gave no history of diarrhea or other symptoms to account for her high temperature. She was not taking any medication. She was pyrexial *(38°C),* tachycardic *(110bpm)* and hypertensive *(160/110mmHg).* Her fundoscopy revealed grade IV hypertensive retinopathy. She had mild pretibial and periorbital edema, with oliguria *(450mL/day).*

**Figure 1:**
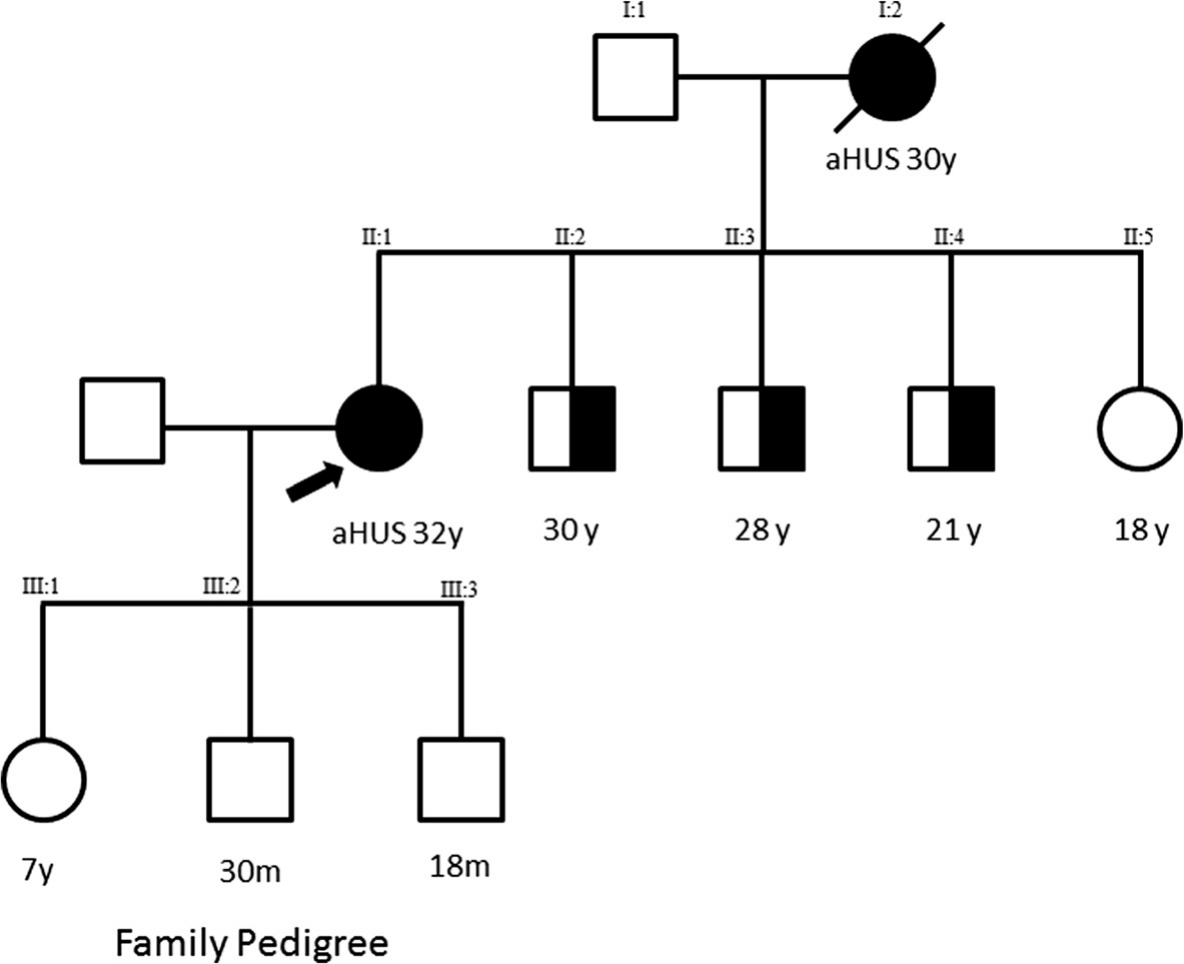
Family pedigree. Family pedigree demonstrating affected individuals and unaffected carriers. Current age or age at presentation of atypical hemolytic uremic syndrome are given in years (y) or months (m). Arrow indicates current patient.

An evaluation of her medical history revealed that she had hypertension, proteinuria and edema during a pregnancy one year previously, which she had successfully completed to term. No laboratory investigations were undertaken at that time. Her mother had died in her early 30s with a clinical picture consistent with a TMA (Figure 1)*.*

Her laboratory evaluation showed microangiopathic hemolytic anemia *(hemoglobin: 6.6g/dL; unconjugated bilirubinemia: 2.1mg/dL; lactate dehydrogenase: 1610U/L (<480U/L); haptoglobin: 10mg/dL (41 to 165mg/dL); schistocytosis: 5%; negative Coombs test; reticulocytes: 3.68%),* thrombocytopenia *(120×10* ^*9*^ */L),* albumin 3g/dL and elevated serum creatinine *(12.65mg/dL)*. Her urinary examination showed +++ proteinuria and the presence of eight to ten erythrocytes and two to three leucocytes; 2.4g protein excretion in a 24-hour urine sample. Viral serologic markers for hepatitis B, hepatitis C and HIV were negative.

A stool culture was negative for Shiga toxin-producing *Escherichia coli*. Her ADAMTS13 activity was within the normal range *(73%),* and she was negative for ADAMTS13 auto-antibodies *(13UI/L).* Antinuclear antibodies *(ANA),* anti-extractable nuclear antigens and anti-double stranded DNA antibodies were negative. Her C4 level was normal *(280mg/L)* but her C3 level was low *(80mg/dL (85 to 200mg/dL)).* An ultrasonography of her abdomen revealed a normal right kidney but left kidney agenesis precluded a renal biopsy.

Microangiopathic hemolytic anemia, thrombocytopenia, renal failure and a likely family history in the setting of normal ADAMTS13 activity led us to the diagnosis of aHUS, and plasma exchange *(*40mL/kg fresh frozen plasma per *exchange)* was immediately instituted. Hemodialysis was also required because of hypervolemia as evidenced by tachypnea, dyspnea, jugular venous distention and intractable hypertension, and pulmonary congestion on her chest X-ray.

**Figure 2:**
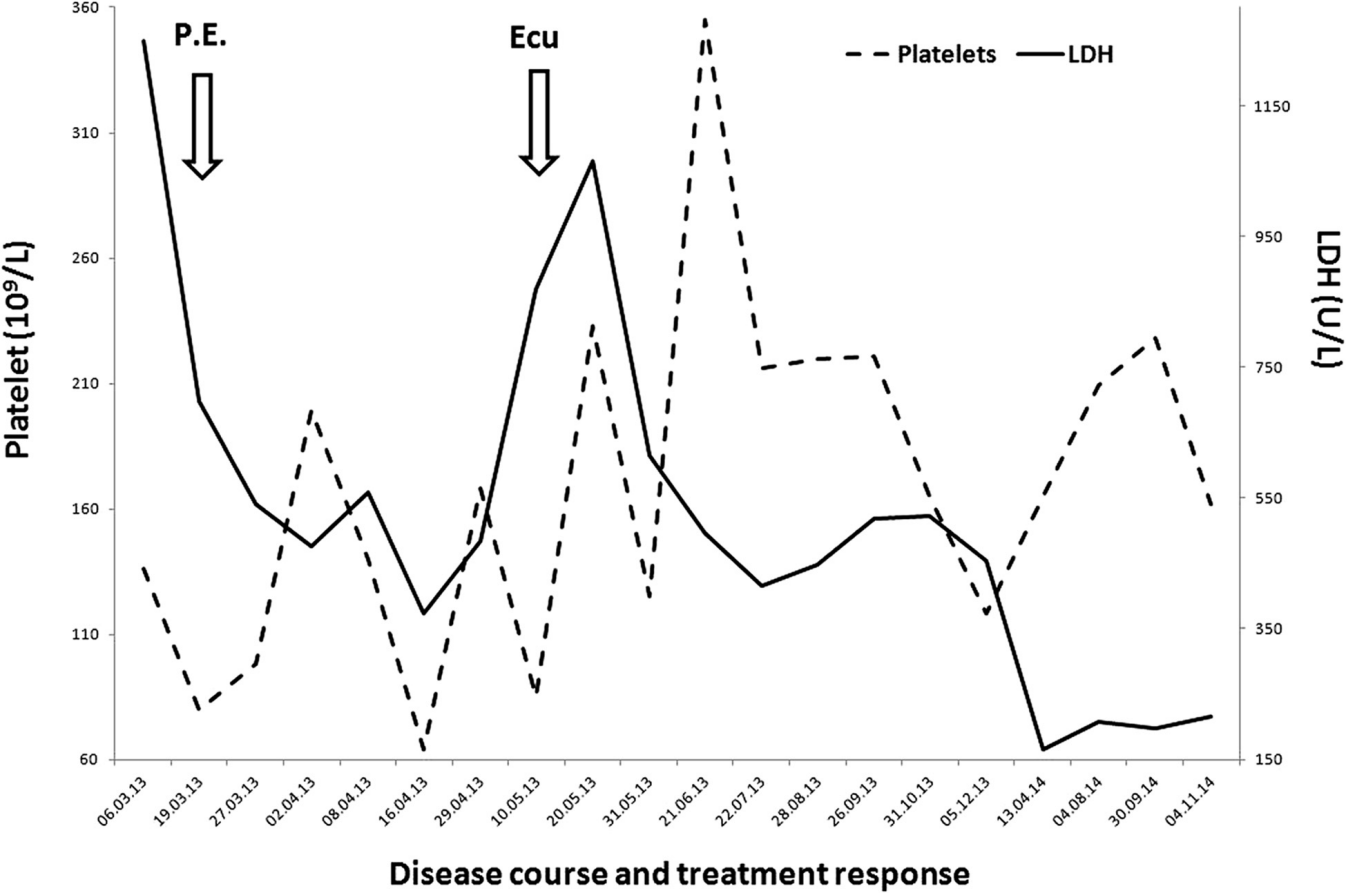
Disease course and treatment response. The timeline from presentation detailing platelet and lactate dehydrogenase (LDH) response to plasma exchange (PE) and eculizumab (Ecu).

Plasma exchange initially improved the biochemical and hematological markers of disease and treatment continued. After 22 sessions of plasma exchange, her LDH level increased to 869U/L and her platelet count fell to *85×10* ^*9*^ */L,* and it was decided to institute eculizumab therapy (Figure 2).

Following vaccination against *Neisseria meningitides,* eculizumab was introduced at 900mg weekly for the first four weeks, followed by 1200mg for the fifth dose one week later and then 1200mg every two weeks thereafter. Her LDH level normalized and no further plasma exchange was required. She had remained dialysis-dependent throughout her treatment, but following two months of eculizumab treatment her renal function improved to the extent that renal replacement therapy was not required. At 20 months into eculizumab treatment her creatinine level was 1.50mg/dL, with no evidence of microangiopathic hemolytic anemia. The drug was well tolerated, with no significant adverse effects.

While management proceeded, genetic analysis was undertaken to screen for predisposing aHUS risk factors as previously described. A heterozygous *CFH* rare genetic variant in exon 21 was found *(c.3454T>A; p.C1152S),* with no other genetic risk factors identified. This resulted in low levels of circulating CFH 0.32g/L *(0.35 to 0.59g/L)*. She was negative for factor H autoantibodies.

A factor H analysis of her father *(I:1)* and sister *(II:5)* revealed that they did not carry the variant, but her three brothers *(II:2, II:3, II:4)* carried the same mutation. The brothers’ renal function was normal and their urine analysis was unremarkable. It is therefore probable that the *CFH* mutation was inherited from her mother, and that the clinical syndrome which led to her death was aHUS; DNA was not available to confirm this hypothesis.

## Discussion

Over the past 15 years, the pathogenesis of aHUS has been demonstrated to be a disease caused by hyper-activation of the alternative complement pathway [1]. Mutations in the complement regulators, *CFH* [4], complement factor I (*CFI*) [5], and membrane cofactor protein (*CD46*) [6], the complement components (*C3*) [7] and complement factor B (*CFB*) [8] have been shown to create a predisposition to aHUS. This ultimately led to the successful introduction of the complement inhibitor eculizumab in aHUS management [3].

In our case report, our patient carried a mutation in *CFH*. Mutations in *CFH* are the most common mutation found in aHUS, accounting for around 25% of cases [1]. The majority of mutations in *CFH* are heterozygous and usually do not result in a quantitative deficiency of factor H. Instead they result in a secreted protein which is unable to bind to, and regulate, complement on endothelial cells, including glomerular endothelium [9]. In our case report, however, the mutation of the invariant cysteine C1152 prevented the formation of the CysI-CysIII disulfide bond with C1109, and thus prevented the formation of a stable protein*.* These predicted structural effects are in keeping with the low plasma factor H levels of our patient.

As is frequently seen, carriers of *CFH* mutations may remain well for many years before presenting with aHUS following an initiating trigger, such as upper respiratory tract infections, fevers, pregnancy, drugs and non *E. coli* diarrheal illnesses [1]. Infectious events, mainly upper respiratory tract infections or gastroenteritis, trigger onset of aHUS in at least half of patients [10,11]. Likewise, in pregnancy-associated aHUS, Fakhouri *et al*. [12] showed that 86% of patients for whom this was a trigger carried a complement gene mutation. In our case report it appears likely that, given our patient’s pyrexia on admission, an unidentified infection was the trigger. It is also of interest that during a previous pregnancy she developed hypertension, proteinuria and edema. Mutations in the complement system have also been described in pre-eclampsia [13].

The inheritance pattern in aHUS is most commonly autosomal dominant, with incomplete penetrance [1]. The proband’s brothers all carry the *CFH* mutation and are at risk of aHUS. They have been counselled to attend for routine hematological and biochemical tests at times of known triggers, such as infection. The proband has elected not to screen her children *(III:1, III:2, III:3)* and as such, they must be considered to be at risk for aHUS, with similar advice given.

Early treatment of aHUS is essential in preserving renal function. It has been suggested that retinal changes, such as the cotton wool spots, flame hemorrhages and papilledema seen in our patient, are rare in HUS and TTP [14]. Retinal examination has been shown to be non-discriminatory for TMAs and should not lead to a diagnosis of malignant hypertension, with a consequent delay in appropriate treatment [15].

Our patient presented with a platelet count *(136×10* ^*9*^ */L)* and creatinine level *(12.65mg/dL)* not suggestive of severe ADAMTS13 deficiency [16]. Her low C3 level, however, was suggestive of complement alternative pathway activity, in keeping with aHUS. Eculizumab is now considered to be the gold standard for management of aHUS. It should not, however, be used until an ADAMTS13 assay excludes TTP. Plasma exchange was therefore initiated immediately, but after an initial response there appeared to be signs of plasma resistance, and eculizumab was introduced. Biochemical markers of microangiopathic hemolytic anemia improved and renal function subsequently recovered despite three months of hemodialysis, as has been reported in other cases [17].

Due to the cost of eculizumab, plasma exchange remains the only treatment available in some parts of the world. In one study from the Italian Registry, 63% of patients with a *CFH* mutation who received some form of plasmatherapy *(plasma infusion or plasma exchange)* had a response to plasma therapy, either complete or partial *(hematological remission with renal sequel)* [11]*.* However, the percentage of complete recovery under plasmatherapy was only 5%, and evolution to death or end-stage renal disease was 77%.

Our patient continues on eculizumab; however, a recent report has suggested that eculizumab may be safely discontinued in some patients, given adequate monitoring [18]. A trial of continuous versus disease-driven eculizumab therapy is required to elucidate the optimal length of treatment.

## Conclusions

In conclusion, we report a case of familial aHUS associated with a *CFH* mutation precipitated most likely by an infectious trigger. We demonstrate grade IV hypertensive retinal changes and severe hypertension as a consequence of aHUS, confirming this as a non-discriminatory sign for malignant hypertension. Although our patient was dialysis-dependent, initial treatment with plasma exchange, and subsequently eculizumab, controlled the disease. After three months on eculizumab therapy, she became dialysis independent. This suggests that, in individuals treated with eculizumab, a minimum duration of three months should be given prior to cessation in the absence of renal recovery.

## Consent

Written informed consent was obtained from the patient for publication of this case report and accompanying images. A copy of the written consent is available for review by the Editor-in-Chief of this journal.

## Abbreviations

aHUS: Atypical hemolytic uremic syndrome
CFH: Complement factor H
CFI: Complement factor I
LDH: Lactate dehydrogenase
TMA: Thrombotic microangiopathy
TTP: Thrombotic thrombocytopenic purpura
